# Role of Catheter Ablation for Ventricular Arrhythmias in Brugada Syndrome

**DOI:** 10.1007/s11886-021-01479-2

**Published:** 2021-04-24

**Authors:** Ronpichai Chokesuwattanaskul, Koonlawee Nademanee

**Affiliations:** 1 Faculty of Medicine, Division of Cardiovascular Medicine, King Chulalongkorn Memorial Hospital, Chulalongkorn University, Bangkok, 10330 Thailand; 2 Bumrungrad Hospital, Bangkok, Thailand; 3 Pacific Rim Electrophysiology Research Institute, Bangkok, Thailand; 4 Pacific Rim Electrophysiology Research Institute, Las Vegas, Nevada USA

**Keywords:** Brugada, Catheter ablation, Sudden cardiac death, Ventricular arrhythmia

## Abstract

**Purpose of Review:**

To discuss the role of catheter ablation in treating life-threatening ventricular arrhythmias associated with Brugada syndrome (BrS), by presenting recent findings of BrS arrhythmogenic substrate, mechanisms underlying ventricular arrhythmias, and how they can be treated with catheter ablation.

**Recent Findings:**

Almost three decades ago when the clinical entity of Brugada syndrome (BrS) was described in patients who had abnormal coved-type ST elevation in the right precordial EKG leads in patients who had no apparent structural heart disease but died suddenly from ventricular fibrillation. Since its description, the syndrome has galvanized explosive research in this field over the past decades, driving major progress toward better understanding of BrS, gaining knowledge of the genetic pathophysiology and risk stratification of BrS, and creating significant advances in therapeutic modalities. One of such advances is the ability for electrophysiologists to map and identify the arrhythmogenic substrate sites of BrS, which serve as good target sites for catheter ablation. Subsequently, several studies have shown that catheter ablation of these substrates normalizes the Brugada ECG pattern and is very effective in eliminating these substrates and preventing recurrent VF episodes.

**Summary:**

Catheter ablation has become an important addition for treatment of symptomatic BrS patients with recurrent VT/VF episodes.

## Introduction

**Fig. 1:**
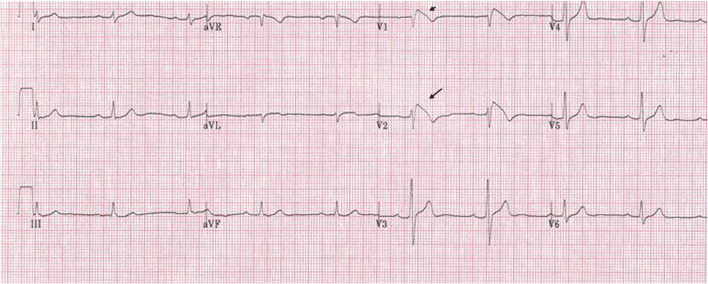
A 12 lead EKG of a BrS patient showing type I Brugada ECG pattern with a signature curved-type ST elevation in V1 and V2 (arrows).

Brugada syndrome (BrS) was first described almost 3 decades ago and was first considered a primary electrical disease manifesting as life-threatening polymorphic ventricular tachycardia/ventricular fibrillation (VT/VF) causing sudden cardiac death (SCD) [1]. BrS is characterized by right precordial lead coved-type ST elevation (Fig. 1) with no overt structural heart disease or secondary factors (e.g., myocardial ischemia, electrolyte imbalance) [2•]. During the first decade, two consensus statements laid out diagnostic criteria, risk stratification, and preliminary genetic aspects of the syndrome [2•, 3•]. Over the last 2 decades, the explosion of seminal research studies shed light on underlying electrophysiological mechanisms of the syndrome—a complex interplay between genetics, myocardial substrate abnormalities, and channelopathy—leading to the development of a new therapeutic modality, catheter ablation of BrS substrates [4•, 5, 6]. The purpose of this review is to discuss the role of catheter ablation in treating life-threatening ventricular arrhythmias associated with BrS, not other aspects of the syndrome. To learn more about other aspects of BrS, we recommend reading several excellent reviews [4•, 5, 6]. Herein, we will describe recent findings of BrS arrhythmogenic substrate, mechanisms underlying ventricular arrhythmias, and how they can be treated with catheter ablation.

## BrS Arrhythmogenic Substrates

It was recently established that right ventricular (RV) epicardium, especially the RV outflow tract (RVOT), is the primary arrhythmogenic substrate site of BrS. Here, abnormal electrograms, defined by low voltage (≤1 mV), prolonged duration (>120 ms), and fractionated potential beyond QRS complex, are omnipresent [7]. These RVOT sites that harbor such abnormal fragmented electrograms were found to have marked epicardial and interstitial myocardial fibrosis, from both biopsy studies of symptomatic BrS patients who underwent direct open thoracotomy ablation for treatment of recurrent VF, and autopsy studies of SCD victims who had a BrS family history. The RVOT sites of the heart were also found to have a diminished connexin-43 expression, resulting in a decrease in gap junction protein connexin-43 when compared to the matched control subjects [8••]. The presence of myocardial and epicardial fibrosis in the RV epicardium of BrS patients likely create a milieu [9], as demonstrated by the study of De Bakker et al. [10], where an electrical impulse must travel in a slow zig-zag conduction manner through the fibrotic areas producing late-fractionated electrogram on RVOT epicardial electrograms, reentrant polymorphic VT/VF, and corresponding BrS type 1 electrocardiogram (ECG) phenotype.

Of significance, our group was the first to show that when radiofrequency ablations were delivered on these fibrotic areas with fractionated electrograms in the RVOT/RV epicardium, Brugada ECG patterns were normalized and effective in preventing recurrent spontaneous VF episodes as well as inducible VF by programmed electrical stimulation [7, 8••]. These observations clearly provide proof that underlying myopathic changes along with their electrophysiological abnormalities likely result in heterogeneity of impulse conduction, conduction delay, and dispersion RV/RVOT epicardium causing reentry as the main electrophysiological mechanism for VF and the signature EKG abnormality in BrS.

## BrS Treatment Before Catheter Ablation

During the first 2 decades of discovering the syndrome, several studies revealed that symptomatic BrS patients—patients who survived previous cardiac arrests, patients with history of unknown syncope/seizure, or patients with observed agonal respiration during sleep—are at high risk for having recurrent VF episodes [11–13]. An implantable cardioverter-defibrillator (ICD) is unequivocally recommended as first-line therapy to prevent SCD in these high-risk patients [3•]. In contrast, there have been no clearly established recommendations for treatment of asymptomatic BrS patients [3•, 14–16]. This was largely due to the lack of strong predictors of VF in the asymptomatic group. Recent studies have reported low but non-negligible arrhythmic events in asymptomatic BrS patients, about a 0.5–1.2% annual incidence, meaning a 12% malignant arrhythmia event at 10-year follow-up [6, 7, 8••]. Some studies recommended that electrophysiology studies be performed to help risk-stratify and decide if ICD is indicated [14–21]. Also, ICD treatment in asymptomatic BrS patients was found to be associated with significant complications, i.e., cardiac implantable electronic device (CIED) implantation, inappropriate ICD therapies, pocket infection, lead fracture, and chronic lead issues [22–24]. As a result, the current guideline does not recommend ICD treatment for asymptomatic patients [3•].

Quinidine is the only anti-arrhythmic drug found to be efficacious in preventing recurrent VF [25, 26]. However, universal inaccessibility, unwanted adverse effects, and patient non-compliance preclude widespread long-term use in most patients [26]. Furthermore, there are scarcity of studies that have a large sample size or randomized clinical trials to critically evaluate safety and efficacy of the drug in symptomatic BrS.

ICD therapy alone does not prevent recurrent VF episodes; the device is merely designed to rescue patients once VF occurs [27]. Numerous BrS patients have suffered from CIED-related issues such as frequent ICD shocks from VT/VF episodes. In the past, many BrS patients had suffered VF storms despite concomitant quinidine treatment that became perilous for such patients to receive intensive care treatment that included isoproterenol infusion, deep sedation, and other anti-arrhythmic drugs including amiodarone. A significant number of these patients succumbed from refractory VF despite medical treatment or suffered hundreds of multiple ICD shocks; a few patients had to undergo cardiac transplantation as a life-saving measure [9]. In short, during the first 2 decades of BrS, there were very limited treatment options for symptomatic patients. Fortunately, ablation of the BrS substrate was developed over the last decade for treatment of the subset of VF storms as well as for symptomatic BrS patients [7].

## Catheter Ablation Treatment

Haïssaguerre and colleagues [29] were the first to attempt catheter ablation to treat BrS patients with recurrent VF. They utilized a VF-trigger ablation approach in symptomatic BrS patients. However, this VF trigger–targeted approach is limited because spontaneous VF-trigger premature ventricular contractions (PVCs) rarely present during the mapping procedure. This poses a major challenge for operators who try to identify the focus of these PVCs triggers, thus precluding its widespread use for treatment of symptomatic BrS.

Subsequently, our group was the first to report the feasibility of epicardial ablations of the VF substrates and that the procedure, utilized in 9 symptomatic BrS patients with recurrent VF episodes, was indeed safe and effective. Since our initial report [7], several studies worldwide have confirmed our findings of epicardial ablation of VF substrates, and the procedure has been increasingly utilized for treatment of symptomatic BrS patients [30–34].

### How to Map Substrate in BrS Patients

**Fig. 2:**
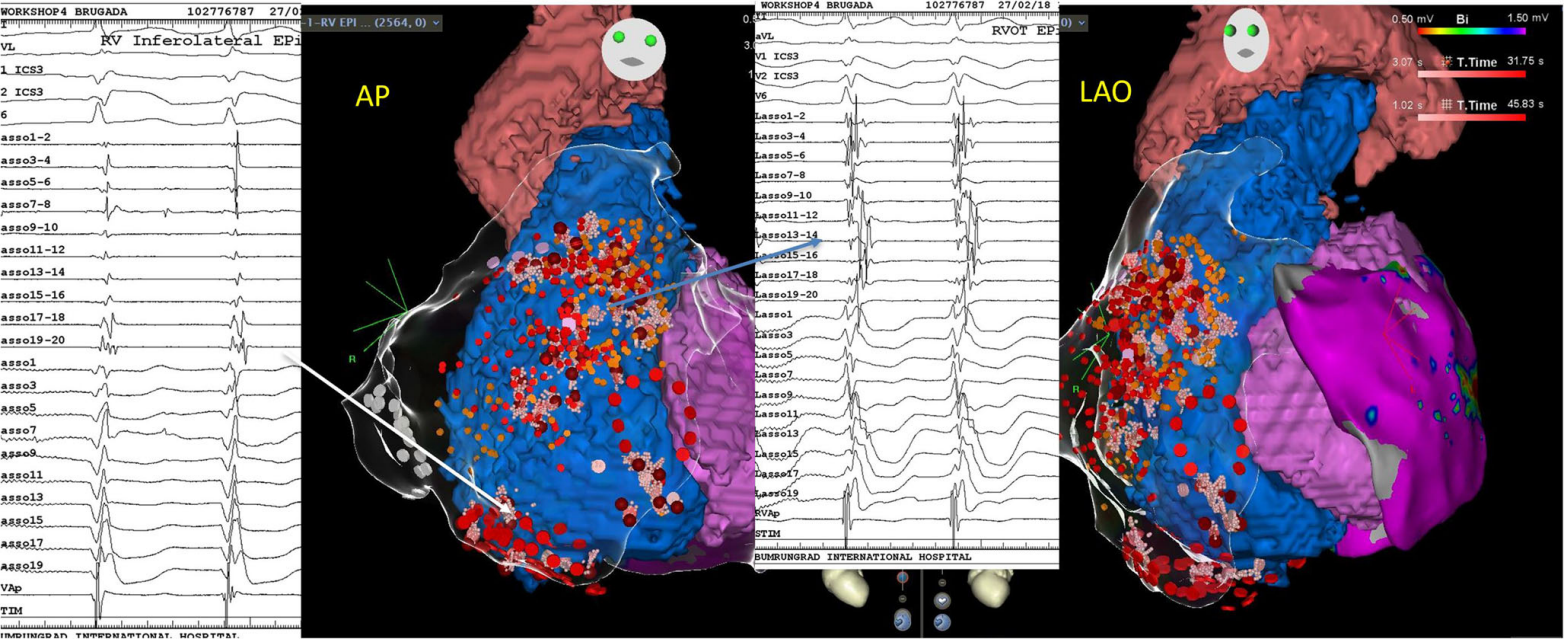
An example of electroanatomic mapping of VF substrates from a BrS patient who had recurrent VF episodes triggering multiple ICD discharges. The electroanatomical maps display areas of fractionated electrograms during sinus rhythm recorded with a 20-pole Lasso catheter in the RV epicardium. The maps are displayed in the anterior posterior (AP) and the left anterior oblique (LAO). Abnormal fractionated potential over the RVOT, RV body, and RV inferolateral are shown in the first and the third insets organized in the following fashion: The top 4 tracings are L1, AVL, V1, and V2 positioned at the 3rd intercostal space (V1ICS_3_ and V2 ICS_3_ respectively) followed with 10 bipolar recordings from the Lasso catheter and the Unipolar recordings from the Lasso poles 1, 3,5,7, 9,11,13, 15, 17, and 19). The first inset is the Lasso catheter recordings from the RV inferior epicardium and exhibits fractionated signals from the bipolar recordings from the Lasso 17–18 and 19–20. The third inset is the lasso catheter recordings from the RVOT epicardium and show abnormal fractionated late electrograms in all bipolar and unipolar recordings. The red dots on the electroanatomical maps represent VF substrates that harbor abnormal fractionated electrograms. Small pink dots are the areas that RF application were delivered. Note that this patient had an extensive epicardial substrates from the RVOT, RV body, and RV inferior wall

**Fig. 3:**
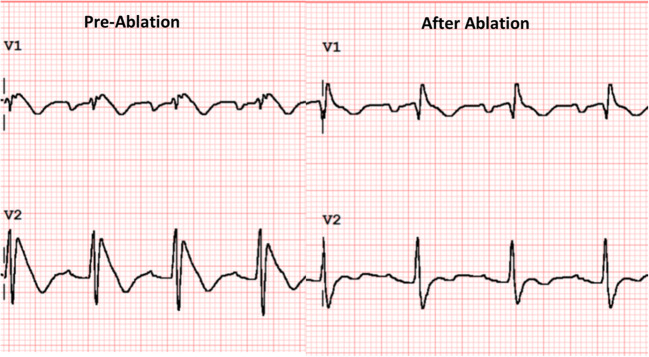
Normalization of Brugada ECG pattern in V1 and V2 after ablation. Both EKGs were taken after ajmaline administration (50 mg)

BrS arrhythmogenic substrate sites have unique electrogram characteristics enabling operators to identify them precisely and easily. To map substrate, one must gain access to pericardial space for epicardial mapping. Both ventricular endocardium and epicardium are mapped with electroanatomical mapping. The mapping system and catheter should be selected based on operators’ preference provided they render high-density electroanatomic mapping. In addition to mapping, image-integration tools, such as CartoUnivu and non-invasive electrocardiographic imaging (ECGI), are beneficial because they [1] provide detailed anatomy and its relationship to the substrate site both during sinus rhythm and during VF [2], identify substrate sites, and [3] prevent collateral damage during radiofrequency application in the proximity of the coronary artery. An example of the map is shown in Fig. 2 which was obtained from a BrS patients with frequent recurrent VF episodes necessitating multiple ICD shocks. The map delineates the targeted area for ablation tagged by the abnormal low-voltage fractionated signals, which comprises multiple components with more than one distinct component, with >20 ms isoelectric segments between peaks of individual components, and long duration (≥70 ms) or late potentials that are beyond the end of QRS complex [28•]. After the ablation, the patient’s EKG has been normalized (Fig. 3) and free of recurrent VF for over 3 years.

### Role of Sodium Channel Blockers in Exposing Subtle Substrate Sites

In our initial report, we performed epicardial RVOT ablation of the first 9 patients without using sodium channel blockade, ajmaline, during the mapping since at that time the Institution Review Board (IRB) did not approve utilization of the drug during the procedure [7]. Subsequently, after the safety of the procedure was established, the IRB granted approval of using ajmaline to further define the substrate sites. Indeed, we found that ajmaline infusion could unmask the subtle substrate sites and further increase the target area to almost twice the size, from 14.3 to 20.4 cm [2•]. A few patients from our initial report experienced recurrence caused by incomplete substrate elimination. These patients required repeat ablation; during this repeated procedure, more substrate areas that were missed during the first procedure were exposed by ajmaline infusion and guided successful ablations [28•]. Pappone and colleagues also reported similar findings with both flecainide and ajmaline that these sodium channel blockades were beneficial in aiding BrS substrates [30, 31]. As a result, a sodium channel blocker has now been included in a mapping protocol to identify all possible substrates.

In the past, endocardial ablation of the substrate sites in the RV was also introduced with promising results [35]. However, the long-term results could not be reproduced among clinical studies. Talib et al. conducted a study demonstrating a stepwise approach of endocardial ablation by searching the VF-triggering area followed by VF substrate ablation endocardial ablation [36]. While they had modest success with VF-trigger ablation, the endocardial approach for elimination of VF substrates yielded a low success rate. This finding was not surprising because virtually all studies found that almost all substrate sites are located in the RV epicardium and not in the corresponding endocardial sites. Thus, it has been our assertion that BrS substrate ablation cannot be carried out successfully without epicardial substrate ablation [37].

### Ablation Protocol and Endpoints

An irrigated-tip catheter with contact force measurement should be used to apply force for ≥5 g before radiofrequency energy is delivered. The power is titrated from 20 to 50 W to achieve ≥10 ohms impedance reduction from baseline. This force and power should provide sufficient acute elimination effect on the target area. Effective lesions were confirmed by rapid disappearance of late/fractionated components and a decline in recorded signal amplitude. The endpoint is a complete elimination of substrate identified after ajmaline infusion over the RVOT and its vicinity. Non-inducible VT/VF and disappearance of the BrS ECG pattern can be used as an adjunct endpoint of ablation.

### Efficacy and Safety of Catheter Ablation for BrS

We have performed ablation on 54 symptomatic BrS patients (median age, 37 years; 1 female). All had ICD for cardiac arrest (*N* = 48) or syncope (*N* = 6). All underwent a percutaneous epicardial substrate ablation with electroanatomical mapping. All patients had BrS substrates in the RVOT epicardium but 25 (47%) also had substrates elsewhere: in the RV exhibiting late-fractionated potentials extended to the RV body (*N* = 14), inferior RV (*N* = 4), and both body and inferior RV (*N* = 7), respectively. After the first catheter ablation, 45 of the 54 (85%) became VF-free. After a repeat CA, 52 patients (96%) became VF-free without anti-arrhythmic drugs (follow-up period = 32 ± 30 months; *p* < 0.01). The main predictor of VF recurrence necessitating repeat ablation is the continuing presence of Brugada ECG pattern and a combined BrS and early repolarization (ER). No major complications occurred, except 1 hemopericardium and 1 pericarditis.

Recent studies from other institutions have confirmed our findings that BrS arrhythmogenic substrates are present in the RV/RVOT epicardium in virtually all symptomatic BrS patients, and catheter ablations of these substrates are safe and effective for prevention of VF recurrences in these patients.

### Combined Syndrome of BrS and ERS

In our patient population in Southeast Asia, approximately 20% have a combined Brugada and early repolarization EKG pattern either in the inferior leads (L II, III, and AVF) or in the lateral leads (V_4_–V_6_) or in both inferior and lateral leads. Several studies in the past decade show that these overlapping features of BrS and ER syndrome (ERS) increase risk of recurrent VT/VF much higher than that pose by BrS alone [38–40]. As indicated above, this subset also presents a daunting task for catheter ablation because the VF recurrent rate, after the first session of ablation, is higher than that of BrS alone. Fortunately, we have now gained more experience in performing mapping and ablation of this subset as we have learned that the combined BrS and ERS patients have larger substrates than BrS alone; in addition to the RVOT substrate sites, the combined syndrome patients commonly have substrates at the RV inferior wall and RV inferolateral areas closed to the tricuspid annulus [41•]. With this knowledge, we have been more proficient in eliminating these substrates yielding better long-term outcomes than the initial encounter with the combined syndrome.

## Conclusion and Future Direction

Better understanding of pathophysiologic mechanisms and underlying pathology of BrS has enabled electrophysiologists to map and identify these substrate sites, which are exclusively present in the RV/RVOT epicardium. These sites serve very well as target areas for catheter ablation. Catheter ablation is effective in eliminating these substrates and preventing recurrent VF episodes. Thus, catheter ablation is an important addition for treatment of symptomatic BrS patients with recurrent VT/VF episodes. In many instances, ablation becomes the sole therapeutic option, especially in the case of drug-resistant BrS or in places where quinidine is not available.

Three key questions need to be answered in the future: (1) Can ablation replace ICD as first-line treatment if all BrS substrates are eliminated? (2) Should asymptomatic BrS patients be treated with ablation? (3) Would extensive substrate ablation over the RV predispose to scar-related reentry monomorphic VT and deterioration of RV function in the long-term? We hope to answer these questions through future randomized clinical trials. Currently, the ablation in BrS for the prevention of VF (BRAVE, Clinicaltrials.gov identifier NCT02704416) study is being conducted; results, available in the next few years, should demonstrate which patients are suitable to be treated by ablation as a curable approach without ICD with long-lasting beneficial effects. At present, we believe that epicardial ablation should be considered the treatment of choice in BrS patients who experience recurrent VF episodes.
